# Netrin-1: Dual Roles in Neuroinflammation and Neurodegenerative Disease Dynamics

**DOI:** 10.1155/ijin/8670048

**Published:** 2025-07-28

**Authors:** Hadiseh Farahani, Ali Ganji, Ghasem Mosayebi, Mohsen Ebrahimi Monfared, Ali Ghazavi

**Affiliations:** ^1^Department of Immunology, School of Medicine, Arak University of Medical Sciences, Arak, Iran; ^2^Molecular and Medicine Research Center, Arak University of Medical Sciences, Arak, Iran; ^3^Department of Neurology, School of Medicine, Arak University of Medical Sciences, Arak, Iran; ^4^Traditional and Complementary Medicine Research Center (TCMRC), Arak University of Medical Sciences, Arak, Iran; ^5^Infectious Diseases Research Center (IDRC), Arak University of Medical Sciences, Arak, Iran

**Keywords:** multiple sclerosis, Netrin-1, neurodegenerative diseases, neuroinflammation, Parkinson's disease

## Abstract

Netrin-1, a central axonal guidance molecule discovered for its role in neuronal development, is also essential in neurodegenerative diseases. Netrin-1 inhibits leukocyte migration and inflammation-related tissue damage outside the central nervous system. Therefore, it can be viewed as a potential biomarker for inflammatory activity in neurodegenerative diseases. Recent studies highlight the dual roles of Netrin-1 in neuroinflammation and neurodegenerative diseases. In the context of neurodegeneration, Netrin-1 demonstrates both protective and harmful effects. This review highlights recent advancements in research regarding the dual roles of Netrin-1 in neuroinflammation and neurodegeneration. We discuss its involvement in protecting the blood–brain barrier (BBB) and regulating immune cell migration and its effects on various neurodegenerative diseases. A greater understanding of the multifunctionality of Netrin-1 could potentially be employed in developing new treatment modalities.

## 1. Introduction

Axons are the primary source of neuronal guidance cues during the central nervous system (CNS) development, spinal cord and peripheral nerve regeneration, and Schwann cell proliferation and migration [1, 2]. These cues encompass a diverse array of family members, including netrins, semaphorins, ephrins, and slits [3]. Netrins are a family of secreted proteins that play crucial roles in axon guidance, cell migration, and inflammation [4]. “Netrin” is derived from “Netr,” a Sanskrit term meaning guidance. This term aptly describes the function of netrins, a conserved family of 70–80 kDa secreted extracellular proteins. These proteins, with sequences similar to the laminin superfamily, play a fundamental role in directing axons toward the ventral midline of the developing nervous system during embryogenesis [5–7]. This evolutionary conservation is further underscored by six renowned netrin genes expressed in mammals (netrins 1–4, G1, and G2) [5]. Netrins 1 to 4 are secreted, while netrins G1 and G2 are plasma membrane proteins that join the membrane by glycosylphosphatidylinositol (GPI) and are not involved in guiding axons or promoting neuronal growth but are known for their function in controlling the formation of synapses [8].

Netrin‐1, a chemokine of significant importance, is expressed in many tissues, spanning from the brain to the lung, heart, liver, intestine, and kidney [9, 10]. Its critical role extends beyond the nervous system, influencing angiogenesis, cell migration, tissue morphogenesis, tumor progression, and regulation of inflammation [11]. The embryonic neural tube expresses it in the floor plate, a specific cell population at the ventral midline [12]. In the adult mammalian, Netrin-1 is secreted by oligodendrocytes, enriching the regulation of cell-to-cell contact [13, 14]. Netrin-1 positively influences the regeneration of peripheral nerves, as well as the proliferation and migration of Schwann cells [1, 2]. Its expression is not limited to the nervous system, as it is also found in various parts of developing and mature mammalian CNS, including the visual [15], olfactory systems, forebrain of adults, and the cerebrum and forebrain of the embryo [16, 17].

Netrin-1 regulates the guidance of neuronal axons in humans mainly through the deleted in colorectal cancer (DCC) and UNC-5 receptors (UNC5A, UNC5B, uncoordinated-5C [UNC5C], and UNC5D), as well as other potential receptors, including neogenin, Down syndrome cell adhesion molecule (DSCAM), adenosine receptor A2b (Adora2b), and integrin subunits [8].

Netrin-1 stands out for its unique dual function. It serves as both a short-range and long-range cue, depending on its proximity to the cellular source [18]. When netrins are close to their source, they exert a short-range effect. Conversely, in long-range cues, the netrin function extends far from the cells secreting netrin [18]. This versatility is evidenced by the long-range cue function of Netrin-1 in the embryonic nervous system [19]. Recent articles present detailed reports on the role of Netrin-1 in neuroinflammation and neurodegeneration disorders [20]. Experiments revealed the essential function of Netrin-1 as a protective or degenerative factor in neuroinflammation [5]. This review presents recent advances in understanding Netrin-1's multifaceted contributions to neurodegeneration and neuroinflammation. By examining its interplay with inflammatory mediators, apoptotic pathways, and disease-specific mechanisms, we aim to clarify its dual roles across neurodegenerative diseases.

## 2. Netrin-1 and Neuroinflammation

Studies of netrin function in the nervous system have revealed a significant role of netrins in regulating cell–cell adhesion and tissue organization. In some cases, after cells migrate initially in response to a source of netrin, it subsequently controls the organization of cell–cell adhesive contacts appropriately. Despite embryogenesis, tissue repair, and direct adult neural stem-cell migration, Netrin-1 plays protective or degenerative roles in neuroinflammation [5].

Neuroinflammation is a complex cellular and biochemical response to brain injury, infection, or neurodegenerative diseases upon exposure to neurotoxic stimulation, ischemic injury, and protein accumulation [21, 22]. Finally, these events lead to the activation of glia, secretion of inflammatory mediators like proinflammatory cytokines and chemokines, recruitment and infiltration of peripheral blood nuclear cells (PBMCs), especially lymphocytes, into the brain, and production of reactive oxygen and nitrogen species [23]. The Netrin-1 gene is identified as a direct transcriptional target of NF-kB. The expression of netrin-1 induced by NF-kB inhibits the proapoptotic activity of its receptors [24]. Netrin-1 preserved normal endothelial function by inhibiting NF-κB activation and the release of TNF-α, IL-1β, and IL-6 [25].

Neurodegeneration is one of the hallmarks of neuroinflammation. It is an inappropriate immune response that leads to the loss of neuronal structure and function in the CNS for various reasons [26, 27]. Neurodegeneration is observed after the immune system insult and causes different CNS problems, called neurodegenerative diseases [23]. They mostly emerge in the mid to late adult life, which notably has a significant impact on older individuals [28, 29], such as multiple sclerosis (MS), Alzheimer's disease (AD), Parkinson's disease (PD), subarachnoid hemorrhage (SAH), and amyotrophic lateral sclerosis (ALS) that result in functional and mental impairments [30].

**Figure 1:**
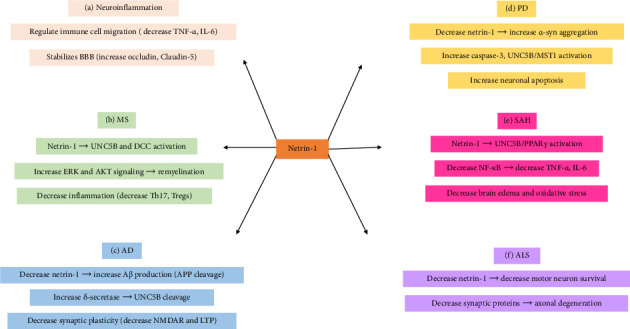
Netrin-1 signaling pathways in neuroinflammation and neurodegenerative diseases. (a) Netrin-1 regulates immune cell migration by reducing TNF-α and IL-6 levels and stabilizes the BBB by upregulating occludin and claudin-5 in neuroinflammation. (b) Netrin-1 stabilizes BBB integrity, activates ERK and AKT signaling to support remyelination, and reduces neuroinflammation by decreasing Th17 and increasing Treg cells in MS. (c) Reduced netrin-1 leads to increased Aβ production via APP cleavage, activation of δ-secretase, cleavage of UNC5C, and impairment of synaptic plasticity through NMDAR downregulation and LTP reduction in AD. (d) Decreased netrin-1 promotes α-syn aggregation and activates caspase-3 and the UNC5B/MST1 apoptotic pathway, leading to increased neuronal apoptosis in PD. (e) Netrin-1 activates UNC5B and PPARγ signaling, which inhibits NF-κB activation, leading to reduced TNF-α and IL-6 levels, decreased brain edema, and oxidative stress in SAH. (f) Netrin-1 deficiency leads to motor neuron loss, decreased synaptic protein levels, and axonal degeneration, contributing to neurodegeneration in ALS. AD: Alzheimer's disease, PD: Parkinson's disease, MS: multiple sclerosis, ALS: amyotrophic lateral sclerosis, SAH: subarachnoid hemorrhage, BBB: blood–brain barrier, APP: amyloid precursor protein, Aβ: amyloid-beta, DCC: deleted in colorectal cancer, LTP: long-term potentiation, NMDAR: N-methyl-D-aspartate receptor, UNC5C: uncoordinated-5 netrin receptor C, MST1: macrophage stimulating 1, UNC5B: uncoordinated-5 netrin receptor B, α-syn: α-synuclein, PI3K: phosphoinositide 3-kinases, AKT: protein kinase B, RAS: rat sarcoma virus, MEK: mitogen-activated protein kinase, and ERK: extracellular signal-regulated kinase.

Because of CNS damage, Netrin-1 is produced by the endothelial vascular system of the blood–brain barrier (BBB) during the neuroinflammatory process. Netrin-1 can inhibit junctional breach and endothelial cell activation in vitro and in vivo [31–33]. BBB integrity is conserved by upregulating endothelial junctional protein expression of Netrin-1. However, Netrin-1 elimination shows disorganized tight junction protein expression and barrier degradation. Eventually, treatment of experimental autoimmune encephalomyelitis (EAE) with Netrin-1 significantly decreased disruption and reduced clinical symptoms of disease severity. Findings have demonstrated that Netrin-1 is a potent regulator of BBB conjunction that defends the CNS against inflammatory conditions (Figure 1) [34].

Different studies assess the role of Netrin-1 in neuroinflammation and neurodegenerative diseases [30, 35]. In this review article, we aimed to introduce some fruitful data about Netrin-1's impact on neurodegenerative diseases so that its positive and negative aspects can be used in future research.

## 3. Netrin-1 and MS

MS is a chronic disease where several pathogenic mechanisms and factors contribute to its occurrence [36]. Neuroinflammation is the most vital pathogenic mechanism that has a significant role at the beginning of the disease. In MS, BBB breakdown and immune cell infiltration into the CNS are critical steps. Inflammatory mediators, which significantly increase in MS patients, form both the mechanisms mentioned above [37, 38]. Alongside chemoattractants that exacerbate the inflammation process, several inhibitory guidance cues also control inappropriate leukocyte migration to CNS [5].

Netrin-1 inhibits leukocyte migration and decreases inflammation-associated tissue injury outside the CNS [39, 40]. Furthermore, the participation of Netrin-1 in EAE mice conserves the stability of BBB by promoting the expression of tight junction proteins such as occludin and claudin-5 [34].

**Figure 2:**
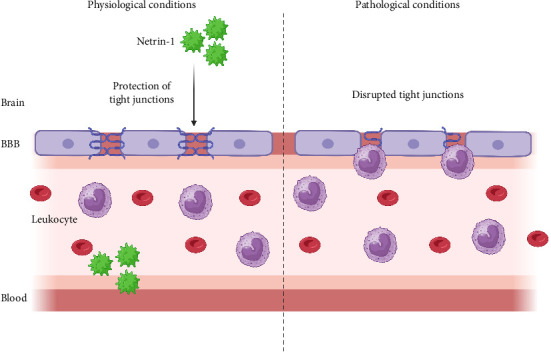
Protective role for netrin-1 in multiple sclerosis. Netrin-1 inhibits leukocyte migration and decreases inflammation-associated tissue injury outside the CNS and conserves the stability of BBB by protecting the expression of tight junction proteins. BBB, blood–brain barrier.

Netrin-1 may limit the immune cells' entrance into the CNS (Figure 2) [34].

It has been revealed that EAE mice had significantly lower Netrin-1 levels and higher TNF-α amounts in serum, spinal cord, and cerebellum compared to healthy control mice. Notably, MS patients declared lower Netrin-1 levels than the control group in serum. The lowest Netrin-1 protein levels were observed in relapsing-remitting MS, significantly lower during the relapse phase. This study revealed that Netrin-1 decreased in EAE and MS patients, primarily throughout relapse, demonstrating evidence of the anti-inflammatory action of Netrin-1 [41]. Truncated forms of Netrin-1, generated by proteolysis, have also been detected in MS lesions. These fragments may have different effects compared to the full-length protein, potentially contributing to the inhibition of oligodendrocyte precursor cell migration [42].

**Figure 3:**
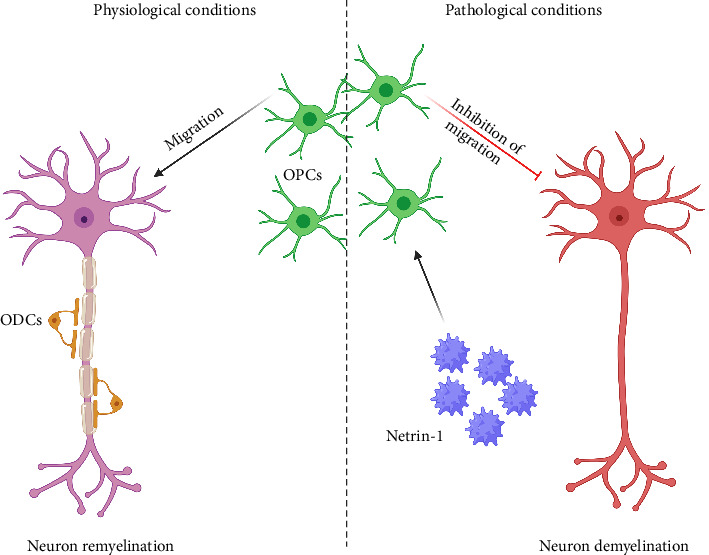
Destructive role for netrin-1 in multiple sclerosis. Netrin-1 in MS plaques can prevent OPC migration and inhibit axon remyelination. OPCs, oligodendrocyte precursor cells; ODCs, oligodendrocytes.

On the contrary, Netrin-1 accumulation via extracellular matrix in lesions significantly inhibits remyelination in MS patients. This inhibitory function of Netrin-1 and its fragments in limiting cell migration and axon growth [43, 44] is a key aspect of the CNS. In this regard, Bin and colleagues showed that Netrin-1 in MS plaques can prevent oligodendrocyte precursor cell migration and inhibit axon remyelination (Figure 3) [17]. After the deterioration of myelin, Netrin-1 is secreted into the MS lesion, where macrophages could phagocytize it or accumulate in the extracellular matrix known as Netrin-1 positive macrophages. Hence, infiltrating these cells into MS plaques may be considered a source of Netrin-1 protein [45]. Therefore, the data indicated that Netrin-1 inhibits the capacity of oligodendrocyte cells to access and repair demyelinated lesions in the pathological process. This understanding of Netrin-1's inhibitory function is crucial for developing strategies to block Netrin-1 function for future treatment of MS [17].

Results indicated that Netrin-1 was significantly enhanced in the serum of MS patients compared to controls and in the blood vessels of perivascular lesions in MS patients and EAE mice, showing increased expressions of Netrin-1 within the brain. Treatment of EAE mice with recombinant Netrin-1 early in the course significantly delayed the onset of the disease and improved disease scores in EAE mice, most likely concerning reductions in inflammatory lesions and proportions of Th17 in the CNS [46].

Repulsive guidance molecule-a (RGMa) belongs to the GPI-anchored proteins with axon guidance function and has been widely implicated in CNS development and pathological processes [47]. Studies have shown the expression of RGMa on the surface of activated microglia [48, 49]. RGMa has an intense inhibitory action on axon regeneration and is also involved in MS neurodegeneration [50]. RGMa is expressed in pathogenic Th17 cells and induces neurodegeneration by binding to neogenin [51]. A possible mechanism is that RGMa induces Akt dephosphorylation in neurons by binding neogenin to Th17 cells [50]. So, indirectly, by binding RGMa to neogenin, Netrin-1 binding to neogenin is inhibited, which can potentially modulate Th17 activation and neuroinflammation [51]. Furthermore, RGMa contributes to the dysfunction of the BBB in endothelial cells, which compromises the integrity of the BBB in MS [52].

The AKT signaling pathway plays an important role in remyelination by ensuring the adequate generation of OPCs and appropriate differentiation of oligodendrocytes through its participation in cell survival and metabolism [53].

The active AKT signaling could help mitigate some adverse effects of inflammation during MS and might be helpful for neuronal survival and myelin repair [54].

Netrin-1 can activate the AKT signaling pathway. Interaction of Netrin-1 with its receptors could result in pull-down and activation of PI3K, which then activates AKT [55].

ERK pathway is a family of MAP kinases whose functional activities involve cell proliferation, differentiation, and survival [56]. ERK signaling has been implicated in many different aspects of oligodendrocyte development, including proliferation, migration, survival, differentiation, and myelination [57]. A number of in vitro studies have pointed to the important role of the ERK/MAPK pathway during OPC differentiation [56]. ERK can be activated by binding via a receptor of Netrin-1, such as UNC5 or DCC, which, in turn, may cause the phosphorylation and activation of ERK. The phosphorylation of Raf (MAP3K) occurs after the Ras family of GTPases is activated. Raf, in turn, phosphorylates mitogen/extracellular signal kinases MEK 1 and MEK 2, which are the immediate upstream activators of ERK1 and ERK2 (Figure 1) [58].

In general, Netrin-1 plays a protective role in MS by preserving BBB integrity by upregulating occludin and claudin-5, reducing CNS inflammation. In EAE mice, recombinant Netrin-1 delays disease onset by suppressing Th17 cells and inflammatory lesions. It activates PI3K/AKT and ERK pathways, promoting OPC survival and differentiation. Moreover, the detrimental roles of Netrin-1 include truncating Netrin-1 fragments (proteolytic cleavage products), accumulating in MS lesions, and blocking OPC migration and remyelination. Full-length Netrin-1 in the extracellular matrix binds OPCs, creating a physical barrier to repair. RGMa binds neogenin, indirectly inhibiting Netrin-1's protective signaling and enhancing Th17-mediated damage.

## 4. Netrin-1 and PD

PD is classically regarded as a motor disorder characterized by the loss of the substantia nigra (SN) dopaminergic neurons and the presence of neuronal Lewy bodies (LB), which are made up of aggregated α-synuclein [59].

Netrin-1 and DCC, the Netrin-1 receptor, are expressed in the adult CNS, particularly in the SN [22, 60]. The midbrain section responsible for controlling movement includes the substantia nigra pars compacta (SNpc) and the substantia nigra pars reticulata (SNpr). The SNpc is primarily composed of dopamine (DA) neurons, whereas the SNpr is composed of GABAergic neurons. DCC shows high levels of expression in DA neurons located in the SNpc [61]. These neurons provide the dorsal striatum with DA and are particularly susceptible to degeneration in PD [61].

In aging, there is a significant decrease in Netrin-1 levels in the brain, especially in PD patient brains, potentially attributed to the loss of dopaminergic neurons, which are a major supplier of Netrin-1 [62]. In PD mouse models, an imbalance of NTN-1 and DCC has been observed as a standard feature in nigral DA neurons. The well-established chemical PD inducer 1-methyl-4-phenylpyridinium iodide (MPP+) has been found to inhibit the expression of Netrin-1 but increase DCC expression in both concentration- and time-dependent manners [63]. Typically, only Netrin-1 is significantly expressed in the SN of healthy adult brains, while α-synuclein is basally present, and their protein levels are inversely correlated. Netrin-1 and α-synuclein have been discovered to interact with each other directly. Netrin-1 blocks α-synuclein aggregation in vitro. Additionally, Netrin-1 deprivation initiates α-synuclein aggregation in cultured primary DA neurons [64]. Hence, the absence of Netrin-1 leads to increased α-synuclein aggregation, potentially playing a role in the development of PD. In experiments with adult mice, selectively removing Netrin-1 resulted in the cleavage of DCC and a notable reduction in DA neurons, ultimately causing impaired motor function in these mice [61].

Chronic constipation is a common symptom that can occur even before the onset of PD [65]. The spread of aggregated α-synuclein-containing LB from the gut to the brain has been suggested as a crucial mechanism in PD development [66]. PD mice exhibit increased intestinal permeability to proinflammatory bacterial products, leading to oxidative stress on the enteric neurons. Research has shown that in the brains and colons of PD patients, there is an inverse correlation between Netrin-1 and brain-derived neurotrophic factor (BDNF) with the inflammatory cytokine-activated transcription factor CCAAT/enhancer binding protein β (C/EBPβ). This result comes from C/EBPβ binding to the promoters of Netrin-1 and BDNF genes to inhibit their mRNA expression [8].

Two potential mechanistic pathways have been investigated in the death of DA neurons caused by Netrin-1 insufficiency: one involves the mammalian Ste20-like kinases 1 (MST1), and the other involves the delta-secretase (asparagine endopeptidase, AEP). The MST1/2 is involved in the Hippo pathway and is critical in controlling tissue growth, cell proliferation, differentiation, and migration in developing organs. Reduction of Netrin-1 activates MST1, which selectively binds and induces phosphorylation of UNC5B on T428 to generate its apoptotic fragment via active caspase-3 in dopaminergic neurons in the SN [62]. Deprivation of Netrin-1 also leads to the downregulation of YAP, a protein involved in scavenging ROS. Both pathways result in dopaminergic neuronal death. Deficiency of Netrin-1 activates delta-secretase (AEP) and caspase-3, which cleaves both α-synuclein and the UNC5C receptor in an age-dependent manner in mice. This results in accelerated DA neuronal loss and PD phenotypes and pathologies. AEP deletion has been shown to rescue these effects. It is worth noting that AEP is highly active in the SNpc regions in human brains with PD, where the DA neurons are mainly located, and Netrin-1 is highly expressed [62]. However, these findings are challenged by previous research, suggesting that Ntn1 is unlikely to be the dominant ligand for the UNC5 family. Additionally, Ntn1−/− mouse embryos exhibit increased expression of DCC and neogenin but no increased apoptosis [67, 68]. The proposed proapoptotic deletion of DCC is instead necessary for dopaminergic neuronal survival during aging [60].

A decrease in Netrin-1 could facilitate the conversion of α-synuclein into the pathogenic pS129-α-synuclein form and may trigger UNC5B phosphorylation through the Hippo/MST1 pathway. These processes ultimately damage dopaminergic neurons (Figure 1) [20].

Altogether, in PD, Netrin-1's protective roles encompass binding directly to α-synuclein, preventing its aggregation into toxic LB in dopaminergic neurons, and maintaining DCC receptor integrity, critical for sustaining DA neurons in the SNpc. The lack of Netrin-1 activates MST1/UNC5B signaling and initiates the death of dopaminergic neurons through caspase-3. A deficiency in Netrin-1 raises δ-secretase (AEP) activity, which cleaves α-Synuclein and UNC5C, speeding up neurodegeneration. Reduced levels of colonic Netrin-1 lead to increased intestinal permeability, which fosters systemic inflammation and the spread of α-Synuclein to the brain.

## 5. Netrin-1 and AD

AD, the most common form of late-onset dementia, is a progressive neurodegenerative disorder that represents nearly 65% of dementia cases in people over 65 years old [69]. Although the FDA has approved lecanemab and similar drugs in America, further evidence is required to confirm their effectiveness and safety [70]. Extracellular deposits of amyloid-β (Aβ) peptide in senile plaques, intraneuronal neurofibrillary tangles, synapse loss, and cognitive decline in the CNS are considered hallmarks of AD [71]. Aβ is derived from the proteolytic fraction of the transmembrane protein known as Aβ precursor protein (APP). The high propensity of Aβ to form oligomers is the most crucial factor for the pathogenesis of AD [72]. APP has been suggested to function in cell adhesion, motility, and synaptic transmission [73]. The APP is fractured by proteases called β-secretase 1 (BACE1), producing Aβ peptide, the chief component of the amyloid plaques related to AD. Netrin binds APP and modulates APP signaling through recruiting APP intracellular domain (AICD)–dependent gene transcription. Moreover, Netrin-1 binding regulates by reducing Aβ peptide generation in brain slices from Alzheimer model transgenic mice [74]. There is an association between declined Netrin-1 expression and increased Aβ levels. The data clarified that brain administration of Netrin-1 in Alzheimer's model transgenic mice might have a strong relationship with an amelioration of the Alzheimer's phenotype [74].

In the other study, a reduction in Netrin-1 levels activates δ-secretase, leading to the cleavage of the UNC5C, as netrin receptor, increased caspase-3 activity, and finally leading to neuronal apoptosis [20].

DCC acts as a potential substrate for BACE1 cleavage in AD. Caspase-3 and BACE1 can cleave DCC, further reducing the amount of full-length DCC protein and contributing to neuronal and vascular damage [75].

A rare mutation in UNC5C enhances a signal that can integrate with APP signaling in AD [76]. A point mutation (T835M) in the UNC5C netrin receptor gene heightens the risk of AD and the susceptibility of neurons harboring various mutation insults [77]. Overexpression of wild-type UNC5C induces low-grade death; however, it is exacerbated by T835M mutation and is deterred by Netrin-1. The neural cell death triggered by the T835M-UNC5C occurs through an intracellular signaling cascade that involves death-associated protein kinase 1, apoptosis signal-regulating kinase 1 (ASK1), protein kinase D, caspases, and JNK/NADPH oxidase. The death signal cascade is merged with ASK1 by the APP, followed by netrin-1 binding to APP and partial inhibition of the death signaling process [76].

Furthermore, long-term synaptic plasticity, including long-term potentiation (LTP) and long-term depression (LTD), is considered the neural basis of the learning and memory process [78]. Netrin-1 guides and localizes synaptic formation by activating the N-methyl-D-aspartate receptor (NMDAR), leading to Netrin-1 secretion in dendrites crucial to synaptic functional modifications. Diminished Netrin-1 levels impair the synaptic plasticity associated with the NMDAR, leading to reduced LTP (Figure 1) [79].

In AD, Netrin-1 is protective by binding to APP, reducing Aβ production and plaque formation. It also enhances NMDAR function, supporting LTP and memory retention. Low Netrin-1 activates δ-secretase, cleaving UNC5C and caspase-3 to induce neuronal apoptosis. The T835M mutation in UNC5C enhances APP-dependent death signaling (via ASK1/JNK pathways), worsening AD pathology.

## 6. Netrin-1 and SAH

**Figure 4:**
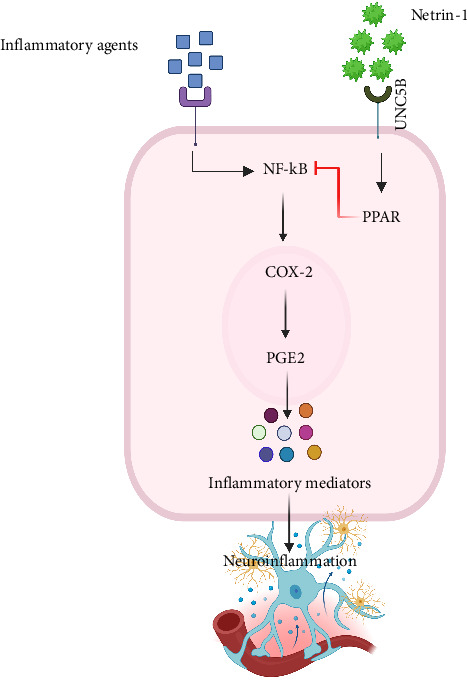
Diagram of the molecular mechanisms of anti-inflammation effects of netrin-1. Netrin-1 activates PPAR, leading to the inhibition of NF-κB and subsequent pathways, and finally suppresses neuroinflammation. NF-κB, nuclear factor kappa light chain enhancer of activated B cells; PPAR, peroxisome proliferator-activated receptors; COX-2, cyclooxygenase-2; PGE2, prostaglandin E2.

Neuroinflammation is a vital mechanism engaged in the pathogenesis of SAH-induced brain injury [80]. Netrin-1 is anti-inflammatory in nonnervous system diseases by preventing neutrophil infiltration [81]. Endogenous Netrin-1 and its ligand UNC5B concentration were increased after SAH. Administration of recombinant human Netrin-1 (rh-Netrin-1) in the SAH rat model attenuated brain edema, ameliorated neurological injuries, and repressed microglia activation after SAH, which were accompanied by peroxisome proliferator-activated receptor gamma (PPARγ) activation, prohibition of NFκB, and decline in TNF-α, IL-6, and ICAM-1, as well as myeloperoxidase (MPO) (Figure 4). Decrease in levels of endogenous Netrin-1 leads to increased production of inflammatory compounds and MPO, resulting in heightened neuroinflammation and brain edema. Furthermore, the destruction of UNC5B and inhibition of PPARγ blocked the preventive effects of rh-Netrin-1. Hence, exogenous rh-Netrin-1 treatment diminishes neuroinflammation after SAH in rats, possibly associated with the UNC5B/PPARγ/NFκB signaling pathway (Figure 1) [82].

In SAH, recombinant Netrin-1 binds UNC5B, activating PPARγ to suppress NF-κB-driven inflammation (TNF-α, IL-6, and ICAM-1). It attenuates microglial activation and oxidative stress markers (e.g., MPO), minimizing post-SAH brain edema.

## 7. Netrin-1 and ALS

ALS is a fatal disease that is the most common form of motor neuron degeneration, a part of the nervous system that controls voluntary muscle movement, with survival of 2 to 5 years from diagnosis. As these motor neurons are lost, the muscles they control become weak and then nonfunctional, thus leading to muscle weakness, disability, and death [83].

It has also been shown that a loss of Netrin-1 is followed by a reduction in synaptic proteins, causing cell death and loss of axons; therefore, the level of Netrin-1 is indicative of neuronal health.

Mutations in the Ub chaperone ubiquilin 2 (UBQLN2) cause X-linked forms of ALS. Genetic screening identified genes involved in the endolysosomal function and axon guidance, such as the UNC-5 netrin receptor, as important regulators of UBQLN2 toxicity. Decreased gene expression of UNC-5 or its partner Dcc/frazzled resulted in reduced neurodegenerative characteristics, such as impaired movement, neuromuscular junction issues, and a shorter lifespan. Future research is needed to understand the impact of UNC5 death signaling on toxicity phenotypes [84].

Netrin-1 sustains synaptic protein levels and motor neuron integrity in ALS, delaying axonal degeneration. On the other hand, reduced Netrin-1 exacerbates UBQLN2 toxicity, disrupting UNC5/DCC signaling and accelerating motor neuron loss.

## 8. Conclusion

Netrin-1 is a multifunctional guidance cue involved in the CNS's development and pathophysiology. During embryogenesis, its bifunctionality as a long- and short-range axon guidance molecule underpins its capacity to coordinate neuronal pathfinding and establish complex neural networks. In addition to developmental functions, the role of Netrin-1 in neuroinflammation and neurodegenerative diseases emphasizes its crucial role in regulating cell–cell interactions and maintaining neurological integrity. Nevertheless, its biological actions are context-dependent, exemplified by its dual roles in neuroprotection—such as BBB stabilization and neuroinflammation inhibition—versus its role in inhibiting regeneration in demyelinating disease. This duality represents the complexity of Netrin-1 signaling and highlights the need to unravel its context-dependent mechanisms.

Netrin-1 binding to its receptors is a complex molecular interaction, with further studies required on implications in neurodegenerative diseases and targets of therapy. In this regard, the expression of Netrin-1 has been associated with disease states, suggesting disturbances in its signaling could play a central role in initiating and developing such disorders. Binding to DCC receptors promotes neuronal survival, as observed in DA neuron preservation in PD. In contrast, engagement with UNC5 can induce apoptosis via pathways such as MST1 kinase in PD or δ-secretase in AD. Notably, proteolytic cleavage of Netrin-1 generates truncated fragments, which antagonize full-length protein activity in conditions like MS, complicating therapeutic targeting. Despite these challenges, Netrin-1 consistently suppresses proinflammatory mediators, including TNF-α, IL-6, and NF-κB, albeit through distinct pathways such as PPARγ in SAH and AKT in MS. These findings highlight the therapeutic potential of modulating Netrin-1 signaling as long as its receptor-specific effects and downstream cascades are clearly defined. Moreover, researchers should pursue to find specific molecular targets of Netrin-1 for neurodegenerative disease. Understanding how Netrin-1 interacts with its receptors and downstream signaling pathways will be crucial for developing targeted therapies. While our knowledge about the anti-inflammatory function of Netrin-1 comes predominantly from studies in model organisms, these findings provide a strong rationale for further investigation of Netrin-1 in human neuroinflammatory disorders. Subsequent research should examine these results in human populations to determine the therapeutic utility of Netrin-1 in neurodegenerative and neuroinflammatory diseases.

In conclusion, Netrin-1 is a promising yet complicated therapeutic target. A deeper understanding of its receptor interactions, signaling plasticity, and context-dependent actions may create a paradigm shift for treating neuroinflammatory and neurodegenerative disorders. Strategic approaches to dissecting its molecular mechanisms, validating findings in human subjects, and developing receptor-specific therapies will be essential to harness its therapeutic potential without causing harmful effects. Such advances can pave the way for new interventions tailored to CNS disorders' intricate pathophysiology.

## Data Availability

The data that support the findings of this study are available from the corresponding author upon reasonable request.
